# Association of chronic hepatitis C with major depressive disorders: irrespective of interferon-alpha therapy

**DOI:** 10.1186/1745-0179-3-22

**Published:** 2007-10-23

**Authors:** Mauro G Carta, Maria Carolina Hardoy, Alessandra Garofalo, Enrica Pisano, Valentina Nonnoi, Gesuina Intilla, Giancarlo Serra, Cinzia Balestrieri, Luchino Chessa, Cristiana Cauli, Maria Eliana Lai, Patrizia Farci

**Affiliations:** 1 Department of Public Health, University of Cagliari, Italy; 2 Liver Unit, Department of Internal Medicine, University of Cagliari, Italy

## Abstract

**Background:**

Mood and anxiety symptoms in chronic hepatitis C (CHC) may be related to the patient awareness of the diagnosis and prognosis, to side effects induced by interferon (IFN)-alpha treatment, as well as to substance abuse. However, the observation of metabolic alterations in patients with CHC has led to hypothesize a direct effect of hepatitis C virus (HCV) on brain function. This study was aimed at elucidating whether CHC is associated with specific anxiety or mood disorders independently of confounding factors.

**Methods:**

Patient cohort: consecutive patients, 135 with CHC and 76 with chronic hepatitis B (CHB). Exclusion criteria: previous treatment with IFN-alpha, co-infection with HCV and hepatitis B virus, infection with human immunodeficiency virus, drug or alcohol abuse, or malignancies. Controls: subjects without evidence of hepatitis randomly extracted from the database of a previous epidemiological study; they were divided into two groups of 540 (332 males) and 304 (220 males) as controls for patients with CHC and CHB, respectively. The psychiatric diagnosis was formulated by means of the Composite International Diagnostic Interview Simplified carried out by a physician according to DSM-IV criteria.

**Results:**

A higher lifetime prevalence of major depressive disorder (MDD) was observed among CHC compared to CHB or controls. The risk of MDD was not statistically different between CHB and controls. Both the CHC and CHB groups showed a significantly higher frequency of panic disorder when compared to controls. No statistical differences were observed in the prevalence of general anxiety disorder and social phobia when CHC or CHB were compared to controls.

**Conclusion:**

The present study provides the first evidence of an association between CHC and MDD, diagnosed on the basis of well-defined international criteria. This association is independent of treatment with IFN-alpha and is not influenced by substance or alcohol abuse. By contrast, anxiety disorders do not appear to be specifically associated with CHC.

## Background

Infection with hepatitis C virus (HCV) is a major public health problem worldwide. Besides the late clinical sequelae of chronic liver disease, such as cirrhosis and hepatocellular carcinoma, a high prevalence of depressive and anxiety symptoms has been reported [1,2]. Patients with chronic hepatitis C (CHC) exhibit worse scores, compared to controls, in health-related quality of life indices, which tend to improve upon successful antiviral therapy [3]. Clinically, these subjects often complain of fatigue, lassitude and impaired memory that do not correlate with the severity of liver disease [4,5]. The high rate of mood and anxiety symptoms in patients with CHC may be related to the patient awareness of the diagnosis and prognosis [6,7], to side effects induced by treatment with interferon (IFN)-alpha, the most effective drug currently available for treatment of HCV infection [8], as well as to past or present substance abuse. However, the observation of metabolic alterations in patients with histologically mild chronic hepatitis C, compared with healthy subjects or patients with chronic hepatitis B (CHB), has led to hypothesize a direct effect of HCV on brain function [9,10].

Until now, no studies have investigated the association between hepatitis C and specific psychiatric disorders. This study was aimed at elucidating whether hepatitis C is associated with specific anxiety or mood disorders diagnosed by structured interviews on the basis of internationally defined criteria. To rule out the confounding factors due to substance (drug and alcohol) abuse or IFN-alpha treatment, patients with such history were not included into the study. The lifetime prevalence of affective disorders was evaluated in a cohort of patients with CHB and CHB, all of whom were drug-free and had never previously been treated with IFN, as well as in a control group of subjects recruited from the general population. It was assumed that the awareness of the disease condition and the stress caused by its uncertain outcome constituted a comparable condition in patients affected by CHB or CHC. The results of our study provide the first evidence of a significant association between affective disorders and chronic hepatitis C.

## Methods

The patient cohort included 211 consecutive treatment-naïve patients with chronic hepatitis B or C seen at the Liver Unit of the University Hospital in Cagliari (Italy) between 2002 and 2004. Subjects who were previously treated with IFN-alpha, co-infected with HCV and HBV, or infected with human immunodeficiency virus were excluded from the study; likewise, subjects with lifetime cocaine, heroine and alcohol addiction, as well as with a diagnosis of malignancies, were excluded. Prior to taking part in the study, all the subjects gave their informed consent. The patient cohort included 135 patients with CHC (83 males and 52 females, mean age 50.7 ± 10.3) and 76 patients with CHB (55 males and 21 females, mean age 44.1 ± 12.1). The diagnosis of CHB and CHC was made according to established criteria. As controls, we studied 844 subjects with no diagnosis of hepatitis, who were randomly extracted from the database of an epidemiological study of health conditions in Sardinia [11]. During this survey, people were asked about their general well being, presence of illness, consultation with physicians and medical tests they underwent both routinely (e.g., work or driver license eligibility) or for health problems.

The selection of the controls was performed from the database of a previous epidemiological survey (1040 subjects) by matching age and sex with the cases using a randomized block design. A block was constructed for each individual constituting the group of cases, that included all eligible age- (± 4 years) and sex-matched controls obtained from the database. Four individuals per block were extracted for each single case, thus implying their automatic exclusion from remaining blocks. The samples of controls for patients with CHB and CHC were extracted independently. Controls subjects were divided into two groups of 540 (332 males and 208 females) and 304 (220 males and 84 females) subjects, who were used as controls for patients with CHB and CHC, respectively.

The psychiatric diagnosis was formulated by means of an interview carried out by the physician using the Composite International Diagnostic Interview (CIDI) in its simplified Italian version [11], a structured interview developed under the aegis of the World Health Organisation (WHO) with the aim of facilitating epidemiological and clinical studies. The computerised algorithm linked to the interview facilitates the formulation of psychiatric diagnoses according to criteria established by DSM-IV (APA 1994 [12]. Cases were interviewed at the Center for Liver Disease and controls in their own homes, as described in previously published studies [11].

### Data analysis

Lifetime prevalence for DSM-IV [12] Major Depressive Disorder, Generalized Anxiety Disorder, Panic Disorder, Social Phobia was calculated in both cases (CHB and CHC) and controls groups; the Odds Ratio association (univariate analysis) for DSM-IV diagnosis (dependent variable) was calculated using the two controls group as "pivot". Statistical significance was calculated using the χ2 test in 2 × 2 tables. Odds Ratio confidence intervals were calculated through application of the method of Miettinen [13].

## Results

The case groups included 135 patients with CHC (83 males and 52 females, mean age 50.7 ± 10.3) and 76 patients with CHB (55 males and 21 females, mean age 44.1 ± 12.1); the control group for CHC included 540 subjects (332 males and 208 females, mean age 50.1 ± 11.2); the control group for CHB included 304 subjects (220 males and 84 females, mean age 44.8 ± 13.5). Data analysis revealed a significantly higher number of lifetime diagnoses of MDD among patients with CHC compared to controls (32.6% vs 12.8%; χ^2 ^= 29.0; p < 0.0001, 1DF, OR = 3.3, CI 95% from 2.1 to 5.1). On the contrary, the risk of MDD was not statistically different between subjects with CHB and controls (17.1% vs 13.8%; χ^2 ^= 0.30, P = 0.58, OR = 1.3, CI 95% from 0.5 to 3.3) (Table 1). Direct comparison between patients with CHC and CHB, after standardisation by sex and age (<45 and >44) with a total of 4 cells and with hepatitis C as a standard sample, demonstrated a significantly higher frequency in the lifetime prevalence of MDD in hepatitis C (32.6% vs 15.1%, χ^2 ^= 6.7, P = 0.009, OR = 2.7, CI 95% from 1.3 to 5.9).

**Table 1:** Lifetime psychiatric diagnoses in a cohort of patients with chronic hepatitis B and in the control group

	Major Depression	Generalized Anxiety Disorder	Panic Disorder	Social Phobia
Chronic Hepatitis	13/76	7/76	6/76	3/76
B	(%)	(%)	(%)	(%)
	17.1	9.2	7.9	3.9
Controls	42/304	38/304	6/304	16/304
	(%)	(%)	(%)	(%)
	13.8	12.5	2.0	5.3
χ^2^	0.30	0.35	5.1	0.81
P	0.58	0.55	0.023	0.37
OR	1.3	0.7	4.2	0.7
CL 95%	0.5–3.3	0.2–2.3	1.2–14.4	0.3–1.5

Regarding anxiety disorders, both groups of patients showed a significantly higher lifetime prevalence of panic disorder when compared to controls (for hepatitis C, 8.9% vs 2.8% in controls, χ^2 ^= 8.9, P = 0.003, OR = 3.4, CI 95% from 1.5 to 7.5, Table 2; for hepatitis B, 7.9% vs 2.0% in controls, χ^2 ^= 5.1, P = 0.023, OR = 4.2, CI 95% from 1.2 to 14.4, Table 1).

**Table 2:** Lifetime psychiatric diagnoses observed in a cohort of patients with chronic hepatitis C and in the control group.

	Chronic Hepatitis C (n = 135)	Controls (n = 540)	c^2^	P	OR	CI 95%
Major Depressive Disorder, No (%)	44 (32.6)	69 (12.8)	29.0	0.0001	3.3	2.1–5.1
Generalized Anxiety Disorder, No (%)	13 (9.6)	49 (9.1)	0.01	0.97	1.1	0.02–504.5
Panic Disorder, No (%)	12 (8.9)	15 (2.8)	8.9	0.003	3.4	1.5–7.5
Social Phobia, No (%)	3 (2.2)	24 (4.4)	0.87	0.35	0.5	0.1–2.1

No statistical differences were observed in the incidence of generalised anxiety disorder (GAD) and social phobia (SP) when patients with chronic hepatitis C or hepatitis B were compared to controls.

## Discussion

The present study provides the first evidence that chronic hepatitis C is associated with major depressive disorders, diagnosed on the basis of well-defined international criteria. Furthermore, our findings demonstrate that this association is independent of treatment with IFN-alpha and is not influenced by substance or alcohol abuse. In fact, these confounding factors were ruled out by the selection criteria of the study. Of note, we failed to find an association between anxiety disorders and hepatitis C. Among anxiety disorders, only panic disorders were more frequent in patients with CHC than in controls, although they were common also in patients with CHB. Thus, our data suggest that anxiety disorders are not a specific consequence of CHC, but rather reflect the stress associated with the awareness of a chronic progressive disease or, in other patient series, alternative risk factors such as drug or alcohol abuse.

The observations reported in the literature about the compromised quality of life among patients affected by CHC, regardless of the severity of the disease [1-3], lead to hypothesize that HCV may exert a direct effect on brain function [4]. Recent studies performed by means of questionnaires concerning the quality of life revealed a high frequency of neurocognitive abnormalities in subjects infected with HCV, both before and during treatment [14,15]. Proton magnetic resonance spectroscopy (H-MRS), a technique that provides information on the brain metabolism, revealed clear anomalies in brain metabolites in vivo in chronic hepatitis C patients [9].

Through application of P300-evoked potentials, a neurophysiological test used to assess the cognitive function, Kramer and co-workers reported that approximately 17% of HCV-infected patients presented abnormal P300 potentials [15]. Modifications of H-MRS and P300-evoked potentials similar to those described above have been widely reported in patients infected with HIV [16-19] a virus that was conclusively shown to infect the central nervous system (CNS). These studies are consistent with the hypothesis that HCV may also be capable of infecting the CNS causing neurological alterations or damage [20]. In spite of the discrepancies present in the literature with regard to the extrahepatic replication of HCV, general consensus seems to exist on the capability of this virus to infect mononuclear cells in peripheral blood and bone marrow [21]. This suggests that monocytes or progenitor cells infected by HCV may introduce the virus into the CNS by means of a "Trojan Horse" mechanism, inducing neuronal dysfunction. This hypothesis is supported by the recent finding of HCV genetic sequences in brain tissue at post-mortem examination [22].

In conclusion, the significant association that we documented between an important psychiatric disorder such as major depressive disorder and chronic hepatitis C in patients who had not been treated previously with IFN and with no history of substance abuse adds ground to the hypothesis that this mood disorder is a direct manifestation of HCV infection. However, our findings are only preliminary and further studies are warranted in order to confirm the hypothesis of an increased risk of affective disorders during chronic hepatitis C infection and elucidate their pathogenesis.
